# How Stress Can Change Our Deepest Preferences: Stress Habituation Explained Using the Free Energy Principle

**DOI:** 10.3389/fpsyg.2022.865203

**Published:** 2022-05-31

**Authors:** Mattis Hartwig, Anjali Bhat, Achim Peters

**Affiliations:** ^1^German Research Center for Artificial Intelligence (DFKI), Lübeck, Germany; ^2^singularIT GmbH, Leipzig, Germany; ^3^Wellcome Centre for Human Neuroimaging, University College London, London, United Kingdom; ^4^Medical Clinic 1, Center of Brain, Behavior and Metabolism, University of Lübeck, Lübeck, Germany

**Keywords:** stress, stress habituation, free energy, goal prioritisation, decision-making

## Abstract

People who habituate to stress show a repetition-induced response attenuation—neuroendocrine, cardiovascular, neuroenergetic, and emotional—when exposed to a threatening environment. But the exact dynamics underlying stress habituation remain obscure. The free energy principle offers a unifying account of self-organising systems such as the human brain. In this paper, we elaborate on how stress habituation can be explained and modelled using the free energy principle. We introduce habituation priors that encode the agent’s tendency for stress habituation and incorporate them in the agent’s decision-making process. Using differently shaped goal priors—that encode the agent’s goal preferences—we illustrate, in two examples, the optimising (and thus habituating) behaviour of agents. We show that habituation minimises free energy by reducing the precision (inverse variance) of goal preferences. Reducing the precision of goal priors means that the agent accepts adverse (previously unconscionable) states (e.g., lower social status and poverty). Acceptance or tolerance of adverse outcomes may explain why habituation causes people to exhibit an attenuation of the stress response. Given that stress habituation occurs in brain regions where goal priors are encoded, i.e., in the ventromedial prefrontal cortex and that these priors are encoded as sufficient statistics of probability distributions, our approach seems plausible from an anatomical-functional and neuro-statistical point of view. The ensuing formal and generalisable account—based on the free energy principle—further motivate our novel treatment of stress habituation. Our analysis suggests that stress habituation has far-reaching consequences, protecting against the harmful effects of toxic stress, but on the other hand making the acceptability of precarious living conditions and the development of the obese type 2 diabetes mellitus phenotype more likely.

## Introduction

Stress habituation occurs in about two-thirds of people when they are repeatedly exposed to the same aversive stimulus (homotypic stressor; Kirschbaum et al., 1995). We define habituators as individuals exhibiting a high tendency for repetition-induced response attenuation (neuroendocrine, cardiovascular, neuroenergetic, and emotional), when repeatedly exposed to the same aversive stimulus (homotypic stressor). Non-habituators have a high tendency not to show such a response attenuation.

In a given study population, repetition of the Trier Social Stress Test (TSST) leads to an attenuation of heart rate and cortisol responses (Kothgassner et al., 2021). However, a closer look shows that only a proportion of the participants habituate. In their classic experiment on stress habituation—which was extremely laborious and thus somewhat unique with five TSSTs on consecutive days—Kirschbaum et al. (1995) observed that habituated participants responded to the first TSST with a steep rise in cortisol but showed virtually no increase in cortisol on the second to fifth repetitions. In contrast, participants who did not habituate showed no attenuation of the cortisol response despite multiple exposures. Those who habituated considered themselves more attractive than those who did not habituate, had higher self-esteem and were less likely to be depressed (Kirschbaum et al., 1995). Of note, stress habituation, which elicits discrete results, i.e., all-or-nothing responses, differs in this respect from response habituation, in which the decrease is usually a decreasing exponential function of the number of stimulus presentations (e.g., withdrawal reflex, postrotatory nystagmus, and galvanic skin response), but both forms of habituation also share commonalities (Thompson and Spencer, 1966; Grissom and Bhatnagar, 2009).

In general, any self-organising system that exhibits sentient behaviour can be seen as an agent. Since stress habituation requires a fully evolved stress system, i.e., sympathetic nervous system (SNS) and hypothalamic–pituitary–adrenal (HPA) axis, we focus here on stress habituation in mammals. To illustrate our proposed mathematical principles underlying stress habituation, we use examples that relate to human agents (work stimulus and social acceptance). This paper explores the question of whether habituation to stress can be explained from first principles.

The free energy principle has become a unifying framework for many types of processes that occur in the human brain and, more generally, living organisms (Friston, 2010). This theoretical framework is grounded in the so-called Bayesian Brain concept and introduced as an account of sentient behaviour, namely, perceptual and active inference (Friston et al., 2006). It has been applied to complex areas such as decision-making, exploration or social cooperation (Schwartenbeck et al., 2013; Hartwig and Peters, 2021). Recent research has also focused on using the free energy principle to better understand conditions such as hallucinations and post-traumatic stress disorder (PTSD; Corlett et al., 2019; Linson et al., 2020). In the free energy formulation, the agent has goal priors that encode the preferred states the agent believes they should occupy; i.e., part of its model of the lived world includes (sub-personal) beliefs about the characteristic states or outcomes they should experience (Friston et al., 2013). Peters et al. (2017) have argued that in a threatening situation, free energy remains high if the agent fails to find a policy that leads to its goal states: if the expected free energy is irreducibly high, this results in a stress response, which in turn allows the agent to leverage the (recognised) state of arousal to resolve the challenges ahead. If a best policy can be found with the help of additional sensory information and enhanced information processing—enabled by stress arousal—and the uncertainty about the policy choice can thus be resolved, the agent experiences ‘good stress’, a sense of mastery and high self-esteem (Kirschbaum et al., 1995; Pruessner et al., 1999, 2005). If no solution can be found in this situation, toxic stress or stress habituation can occur. While toxic stress results in intermittent or permanent hyperactivity of SNS and HPA-axis, stress habituation leads to attenuated stress responses.

Since the free energy principle explains a range of phenomena in neuroscience, we ask here whether it can also explain the process of stress habituation. For PTSD, a connection to the free energy principle and to the shape of goal priors has already been formulated but without describing the process of change (Linson et al., 2020). Based on the free energy principle, both PTSD and stress habituation can be viewed as processes that change the shape of goal preferences but with changes occurring in the opposite direction: in PTSD, the precision of the goal-preference probability distribution is increased, in stress habituation, it is decreased.

This paper is structured as follows: First, we introduce decision-making under the free energy principle in general (Section “Free Energy Principle and Decision-Making”). Afterwards, we define stress, discuss stress habituation and show how stress can be caused in an agent under the free energy principle in Section “Stress and Stress Habituation.” In Section “Introducing a Habituation Prior Into the Model,” we introduce a general model that allows for stress habituation through changes in goal priors and give two examples in Section “Stress Habituation in the Context of Work Stimulus Preferences and Social Acceptance.: In Section “Consequences of stress habituation,” we discuss the pathophysiological implications. We conclude in Section “Conclusion and Outlook” with an outlook for further research.

## Free Energy Principle and Decision-Making

The free energy principle is a formalisation and extension of the influential observation that living organisms are, by definition, self-organising systems (Schrödinger, 1956): they maintain a homeostatic balance by returning consistently to a limited set of states. When they cease to self-organise, they die. Mathematically, it emerges that all self-organising systems (look as if they) are driven to minimise a quantity called *free energy* (Friston et al., 2006). This mathematical consequence identifies (variational) free-energy minimisation as the fundamental imperative of all self-organising systems, from the simplest (e.g., a thermostat or a virus) to the most complex (e.g., a human or even civilisations of humans)—this is the free energy principle.

Active inference is a process theory that applies this principle to sentient behaviour, specifying that homeostasis can be maintained either by changing in response to the environment (adaptation) or—crucially—by changing the environment (action/agency). In both cases, the imperative is a convergence between the environment and the agent’s generative model of that environment (Friston et al., 2010; Parr and Friston, 2018, 2019). An agent’s generative model is a probabilistic account of the causes of incoming sensory data (e.g., a flashing light or a noxious smell). For more sophisticated systems, this model also represents sequences of actions through time (or *policies*), from which the agent must select in order to minimise *expected* free energy. Policy selection, according to the process theory of active inference, is what underwrites decision-making and, indeed, all agentic behaviour. For context, active inference originated in the realm of human neuroscience, so the terminology and mathematics used here derive from Bayesian statistics, following from the Bayesian brain hypothesis (Rao and Ballard, 1999; Knill and Pouget, 2004).

The agent is therefore said to hold (sub-personal and Bayesian) beliefs about the hidden states of its environment that maximise the model evidence or minimises surprisal, also often referred to as surprise (Friston et al., 2006). We follow Buckley et al. (2017) in the use of surprisal to ‘avoid confusion with the common sense meaning of surprise’. Under the free energy principle, free energy is an upper bound on surprisal. In this paper, we denote the hidden states of the environment with *s* and the agent’s observations as *o*. The agent has an approximate representation of the states in the world denoted as *q*(s), which approximates the real posterior distribution over the hidden states given the observation *p*(s|o). The agent has the opportunity to influence its own perception (or ‘actively sample’ sensory information; Parr and Friston, 2018) by varying control states (e.g., a variable to control its head movement). Modulation of control variables yields observable actions in the real world (e.g., the head is actually turning). The choice of future control variables—policy selection—is encoded in the policy *π*. We assume that the agent has a limited number of discrete choices for its policy selection. In each time step, the policy is selected that has the lowest expected free energy. Policy selection and thus decision-making can be modelled as minimisation of expected free energy. Technically, the expected free energy is the free energy of observable outcomes and beliefs about states of the world, expected under the outcomes predicted given a course of action or policy. The free energy itself can be expressed as a combination of complexity and accuracy:


$$\begin{array}{c} F=complexity-accuracy\\ =KLD\left[q\left(s|\pi \right)||p\left(s\right)\right]-\underset{s}{\sum^{\text{​}}}q\left(s|\pi \right)\log\left(o|s\right) \end{array}$$

The complexity term represents the divergence between the prior and posterior beliefs. The greater this divergence, the more extreme the belief update the agent will have to make to account for the new information. In other words, a model with greater complexity is generally in danger of overfitting, with a higher probability of the agent having to significantly change its beliefs to explain new observations (i.e., lower predictive power). The ordering in the Kullback–Leibler-divergence (KLD) follows the definitions of the free energy principle. The accuracy is just the expected log likelihood of observed outcomes, given beliefs about their causes (e.g., the amplitude of residual or prediction errors in a statistical model). When complexity and accuracy are evaluated in the future, they become ‘risk’ and ‘ambiguity’. Risk is now the divergence of anticipated states of affairs given a particular policy from the prior preferences or goal prior p(s), which models the agent’s beliefs about its characteristic or preferred states. Similarly, the expected inaccuracy becomes ambiguity; namely, the degree to which a particular state of affairs generates ambiguous outcomes. In short, agents are compelled to choose policies that minimise the risk of diverging from goals, while avoiding ambiguous situations.

Friston (2010) frames goals as ‘prior expectations that an action is obliged to fulfil’. The most primitive goal priors, it could be said, are specified by natural selection (Friston, 2010). The classic example is a fish that likes (its goal prior is) to be in the water, as this is the environment its physiology (fins and gills) is adapted for. The fish existentially prefers survival to death, so these physiological constraints limit the actions of the fish to those that keep it within a range of states it can survive in. If the fish could plan, then it would choose those policies that avoid leaving water. When the goal prior cannot be fulfilled, the risk (expected complexity) term increases, resulting in the agent expecting a higher free energy. The fish who finds itself out of water would indeed experience a high free energy. For humans, we can think of these primitive goal priors as analogous to the lowest rung of pyramid of needs of Maslow (1943). In sense of Maslow (1943), humans also have higher rung goal priors, such as social acceptance and self-actualisation.

The ‘states that agents believe they should occupy’ are thought to be represented in regions like the ventromedial prefrontal cortex (vmPFC) and the orbitofrontal cortex (OFC; Bechara et al., 2000; Gottfried et al., 2003; O’Doherty et al., 2003; Roesch and Olson, 2004; Barron et al., 2015). These regions occupy a deep or high hierarchical position in the Bayesian brain. They play a key role in defining the expected value (i.e., expected free energy) of future outcomes. These goal or prior preferences provide a point of reference for goal-directed behaviour. Behavioural experiments provided evidence that beliefs about goal states are encoded in the vmPFC as sufficient statistics (mean and variance) of internal probability distributions over the states of the world (Lebreton et al., 2015). Interestingly, then, each goal prior is accompanied by a second-order valuation (a confidence estimate), which is also evinced in vmPFC activity (Lebreton et al., 2015).

A very special belief about goal states is whether one wants to hold on to the other goal priors (social recognition, pecuniary goals, housing situation, etc.) under all circumstances or whether one is prepared to give them up, at least in part. In this paper, we propose that stress habituation is based on a broadening (i.e., decrease in precision) of goal preferences, so that states that previously appeared unacceptable henceforth become more acceptable. In other words, stress habituation ‘relaxes’ the degree of commitment to prior preferences. We will refer furthermore to anatomical and functional data that show the representation of goal states and the process of stress habituation are co-localised. The next section concerns the consequences of high free energy and how we respond to it.

## Stress and Stress Habituation

### Stress

Stress, as redefined by Peters et al. (2017), is grounded in the concept of uncertainty. Accordingly, people ask themselves, on certain occasions, the following question: ‘What policy should I select to safeguard my future physical, mental and social wellbeing?’ Whoever is uncertain in their answer experiences ‘angst’, and who on top of that only has high-risk policies available, experiences ‘stress’. In the context of stress, uncertainty means that no matter what policy the agent chooses, they are uncertain about what will happen sooner or later, i.e., they expect, on average, greater surprises. In technical terms: all plausible policies have high expected free energy; i.e., all policies entail a high degree of ‘risk’. Heuristically, expected free energy is the expected surprise associated with a policy; either in terms of uncertainty about states of affairs (i.e., ambiguity) or the kind of surprise that accompanies the violation of preferred outcomes or goals (i.e., risk). Under this formulation, physical, mental, and social well-being are exactly what should be encoded in the agent’s goal priors. Simply said, a high expected free energy, for all policy options, characterises a situation where no policy can realise goal states, thus engendering angst, and ultimately, stress (Peters et al., 2017).

There is evidence that some of our model parameters are represented in mammalian brains. The anterior cingulate cortex (ACC) is involved in assessing policy risks (Paulus et al., 2002; Feinstein et al., 2006; Behrens et al., 2007; Sarinopoulos et al., 2010; Karlsson et al., 2012; Liljeholm et al., 2013). Thus, the ACC is in a position to detect a high expected free energy. When all policies have a high expected free energy, the precision of beliefs about policies is necessarily low. This means that the question ‘What should I do?’ cannot be answered unequivocally. Thus, if the expected free energy is high for all policy options, we suppose that the ACC neurons disinhibit a stress response. The rationale behind this is that the ACC sends strong and functionally relevant projections to the amygdala (Jhang et al., 2018), the hierarchical neuroendocrine stress response involves activation of the amygdala, hypothalamic nuclei, sympathetic nervous system, hypothalamic–pituitary–adrenal axis, and feedback of glucocorticoids to all levels (Peters et al., 2007; Weidenfeld and Ovadia, 2017). The agent’s experience during stress arousal includes symptoms such as increased vigilance, tension, trembling, sweating, palpitations, inner unrest, physical discomfort, anxiety, and sadness (Peters et al., 2013). At the neurobiological-neuro-statistical level, the stress response involves the activation of an uncertainty resolution programme, with the goal of minimising free energy in the future. To achieve this, the uncertainty resolution programme revises the precision afforded various (Bayesian) beliefs,^1^ with the objective of restoring the precision of beliefs about policies. The uncertainty resolution programme has the potential to find out that a policy in the agent’s repertoire is promising after all or to creatively search for a new policy that eventually appears promising. If the programme is successful, the most plausible policy can be selected which, in the sense of active inference, is most likely to minimise free energy.

For completeness, it should be noted that the precision of beliefs about policies can also be low if every option has a low expected free energy, i.e., is relatively risk-free. This is the case, for example, in daily life when an agent is faced with a very large choice, often referred to as choice overload. Here, too, the decision of what to choose is difficult. In such an ambiguous situation, the agent feels unsure about policy selection and experiences angst and discomfort. The resolution of this discomfort—say by the appearance of a conditioned stimulus—appears to be accompanied by phasic dopaminergic discharges (Schultz, 2007; Fiorillo et al., 2008; Schultz et al., 2008; Friston et al., 2014, 2017; Schwartenbeck et al., 2015). As long as the risk is low, however, this situation is existentially harmless, and the agent anticipates that their preferences can be fulfilled irrespective of the particular policy they commit too.

Examples of stressful situations can be manifold, and each person has their own stress biography. The classic choice in stressful situations is the fight-or-flight decision. Stress also arises, for example, in a ‘stay-or-go’ conflict at work, where the person is threatened by either persistent bullying or unemployment. Both options, stay or go, result in major violations of goal expectations and are associated with a high risk or expected complexity.

From a neurobiological and neuroenergetic perspective, the stress response comprises three sub-responses, as shown in our earlier integrating work (Peters et al., 2017): first, a hypervigilant arousal state that allows for higher rate of information processing (bits/second; Berridge and Waterhouse, 2003; Aston-Jones and Cohen, 2005; Harris et al., 2012), second, activation of the sympathetic nervous system (SNS), which supplies the brain with additional energy from the body (glucose, ketones, and lactate), to meet the brain’s higher energy needs due to increased information processing (joules/bit; Madsen et al., 1995; Hitze et al., 2010) and third, the release of glucocorticoids that control what is and what is not learned from the stressful experience for making better predictions in the future (Pavlides et al., 1995; Maggio and Segal, 2007, 2009). All three of these responses reflect an increase in the precision or rate constants of neuronal message passing in cortical hierarchies, manifest in terms of the rate of evidence accumulation, the accompanying metabolic cost and learning rates. However, these stress-related increases come with side effects.

Prolonged glucocorticoid secretion can have adverse effects on brain and body (McEwen, 1998). In fact, toxic stress has been confirmed to cause myocardial infarction (Orth-Gomér et al., 2009; Gulliksson et al., 2011) and increase the risk of depression, memory loss and type 2 diabetes mellitus (Heraclides et al., 2009). As already mentioned, in the best-case scenario, an agent can use the stress arousal state to find an alternative solution to the problem by entertaining new policies, e.g., by withdrawing from the confrontation or creatively engaging a novel policy. But in the worst-case scenario, if no clear option can be found, there are two further possibilities. One possibility is that the agent remains in a state of toxic stress, in which glucocorticoid levels are constantly or intermittently elevated, ultimately leading to detrimental effects on physical and mental well-being (McEwen, 2012). Another option is stress habituation, in which the glucocorticoid response is reduced for certain homotypic stressors, but the ability to provide a hormonal response for other different stressors (heterotypic stressors) is preserved (Hill et al., 2010b).

### Stress Habituation

Anatomical localisation associated morphological changes and endocannabinoid signalling of stress habituation have been intensively studied in rodent experiments. Stress habituation is controlled by brain regions such as the medial prefrontal cortex (mPFC) permitting or disrupting HPA-axis activity (Weinberg et al., 2010). Habituation to stress is accompanied by structural changes in the mPFC. For example, repeated brief restraint, a treatment that generally induces habituation, results in retraction of the basal dendrites of the mPFC (Brown et al., 2005). Prolonged restraint stress leads to dendritic retraction and loss of spines in the mPFC (Cook and Wellman, 2004; Radley et al., 2006, 2008), and chronic immobilisation increases branching of GABAergic interneurons in the PFC (Gilabert-Juan et al., 2013).

Endocannabinoids (eCBs) account for both the ability of glucocorticoid signalling in the mPFC to terminate stress-induced-HPA-axis response and to establish stress habituation. Termination of stress-induced-HPA-axis response involves mobilisation of eCBs in the mPFC (Hill et al., 2011b). After resolution of stress, the cannabinoid-1-receptor (CB1R) pathway contributes to the return of serum glucocorticoid levels to normal. CB1Rs are present on GABAergic terminals and inhibit GABA release at synapses with principal neurons within layer V of the prelimbic region of the mPFC. Glucocorticoids suppress GABA release in the mPFC *via* an endocannabinoid mechanism (Hill et al., 2011b).

Habituation to stress also involves endocannabinoid signalling (Hill et al., 2010a). The endocannabinoids act in a bisynaptic process (Hill et al., 2011b) that is referred to as ‘depolarisation-induced suppression of inhibition’ (DSI; Pitler and Alger, 1992). As Peters and McEwen (2015) have proposed, this DSI process acts as a switch, with the ON position enabling habituation and the OFF position not. Three neurons are involved in this bisynaptic process: a presynaptic mPFC principal neuron, its postsynaptic glutamatergic counterpart and a GABAergic interneuron whose terminals contact the former presynaptic principal neuron. A homotypic stressor stimulates a principal mPFC neuron to release glutamate, which activates the postsynaptic neuron and, if the stimulus is strong enough, prompts the postsynaptic neuron to retrograde release large amounts of endocannabinoids. The eCBs, in turn, inhibit the GABAergic interneuron, which inhibits the presynaptic principal neuron. Thus, eCBs exert a reinforcing, i.e., disinhibitory, effect on the presynaptic principal neuron. In this way, only strong stimuli can turn the DSI switch into the ON position.

During severe stress, elevated glucocorticoid concentrations enhance endocannabinoid production in the postsynaptic neuron. Tagged by putting the DSI switch in the ON position, the presynaptic principal mPFC neurons are prone to undergo plastic changes at their GABAergic synapse through the induction of inhibitory long-term depression (I-LTD; Hill et al., 2011b). After habituation, especially by the I-LTD, the DSI switch would remain in the ON position, so that this mPFC neuronal ensemble enters a new state fixed by synaptic plasticity. Under this model, habituation is induced only when two factors are present simultaneously: the homotypic stressor must represent a strong stimulus and the glucocorticoid response must be strong. Therefore, habituation to stress manifests itself as a specific form of synaptic and morphological plasticity, in the induction of which not only glucocorticoids but also endocannabinoids play a key role (Patel and Hillard, 2008; Hill et al., 2010a).

To distinguish individuals who tend to habituate from those who do not, the mechanical elements involved in the DSI switch are promising candidates. Among them are the glucocorticoid receptors (Malcher-Lopes et al., 2008; McKlveen et al., 2013), the cannabinoid receptors (Fride et al., 2005; Hill et al., 2011a) and the endocannabinoid degrading enzyme fatty acid amide hydrolase (Hill et al., 2013). Different characteristics in these candidates are likely to contribute whether an individual is a habituator or a non-habituator. Interestingly, polymorphisms in the glucocorticoid and endocannabinoid receptor gene and in the fatty acid amide hydrolase gene have been found associated with several differences in body shape (van Rossum and Lamberts, 2004; Benzinou et al., 2008; Frost et al., 2010; Harismendy et al., 2010). The relationship between habituation and body shape will be discussed in more detail below.

How do the conventional and our novel view of stress habituation explain the anatomical and functional data just cited? According to the conventional view, the plasticity acquired through inhibitory long-term depression can lead to information about the homotypic stressor not being transmitted to the amygdala, so that a stress response does not occur. Our novel view of stress habituation is informed by experimental results showing that where the goal states are encoded, namely, in the vmPFC and OFC (Bechara et al., 2000; Gottfried et al., 2003; O’Doherty et al., 2003; Roesch and Olson, 2004; Barron et al., 2015), the process of stress habituation is also localised; to be more precise, stress habituation is located in a subdomain of the vmPFC, that is, in the mPFC (Weinberg et al., 2010). Therefore, we introduce here the concept that in the mPFC, first, the inhibitory long-term depression induced by habituation flattens (i.e. reduces the precision of) probability distributions that encode goal priors and, second, that in the ACC this leads to a new risk assessment of the agent’s available policies. If decreasing the precision of goal preferences reduces the risk of all available policies, there will be no—or only a small—amygdala activation and thus an eliminated or attenuated stress response. Our view is further supported by experimental evidence showing that habituation to repetitive stimuli, as seen in the well-established mismatch negativity paradigm, rests upon hierarchical predictive coding (Wacongne et al., 2012; Ramaswami, 2014).

### Stress Habituation and the Problem With Fixed Goal Priors

When we connect the decision-making under the free energy principle with the phenomenon of stress habituation, we need a way to differentiate between non-habituators and habituators. In the stressful situation, there is no policy *π* for which the predictive posterior *q*(s|*π*) is close to the goal prior *p*(s). In the described scenarios, the policies and the corresponding posterior distributions do not change. In the stay-or-go conflict, both habituators and non-habituators have the same policy choices and possibly the same preferences (showing the same initial stress response), but they react differently when being exposed again. If the posterior is not changed, another option is to change the goal prior. Here, we assume that the goal priors are encoded by synaptic connection strengths in specific (e.g., medial prefrontal cortex) canonical microcircuits, such that, in extreme stress situations, a physiological process may modify goal priors. This Bayesian belief updating of priors is an emergent property of free energy minimisation and reflects the hierarchical generative models used for belief updating, where priors become empirical or learnable priors (Sajid et al., 2021).

In the next section, we look into how to setup a decision-making model including the option for habituation. Peters et al. (2017) have previously suggested that habituation might be connected to an influence on the goal prior as a last resort in stressful episodes. As described above, the goal prior has a direct connection to the preferred states of the agent. Therefore, changing the goal prior comes at a significant cost of compromising preferred states. To prevent the agent from making these sacrifices carelessly, some kind of internal barrier or threshold needs to be present, which allows habituation only as a last resort. Next, we will focus on how habituation might be mathematically implemented into decision-making using the free energy principle.

## Introducing a Habituation Prior Into the Model

Given the classical experimental findings on stress habituation mentioned above (Kirschbaum et al., 1995), we model stress habituation as a discrete process in which the agent either habituates or not. The differences between habituators and non-habituators are encoded within the agents. Some (but not necessarily all) characteristics of the goal prior are affected by stress habituation. The stress habituation is a result of optimising free energy.

We represent the habituation in a habituation variable *h*. If *h* = 1, the agent habituates; if *h* = 0, the agent does not habituate. In order to encode different tendencies for agents to habituate we introduce a habituation prior *p(h)*. It is $p\left(h=1\right)=\hat{h}$ and $p\left(h=0\right)=1-\hat{h}\text{.}$ We call $\hat{h}$ the habituation tendency. A zero tendency $\hat{h}$ stands for an agent that cannot habituate no matter the stress level it is experiencing. A higher tendency makes habituation more likely, as we will see in later simulations. We use the new habituation prior to extend the existing goal prior distribution with $p\left(s\right)=p\left(s|h\right)p\left(h\right)\text{.}$ The conditional distribution *p*(s|h = 0) is encoding the agent’s initial goal prior, whereas the $p\left(s|h=1\right)$ is encoding the agent’s goal prior after habituation. To explain the transition (and the difference) between the two distributions, we introduce a parameter $\theta_{h}$ that has a statistical influence on prior distribution. In general, $\theta_{h}$ can be any of the sufficient statistics for the distribution but one example that we will use later is the variance of the distribution. The parameter $\theta_{h}$ can be changed only if the agent habituates. Analogue to the approximate posterior distribution that depends on the policy choice $q\left(s|\pi \right)$, we also need a posterior *q*(h) that reflects if the agents habituate or does not habituate. Introducing these distributions and variables in the risk term of the expected free energy results in:


$$Risk=KLD\left[q(s|\pi ,h)q(h)\mathrm{||}p_{\theta_{h}}(s|h)p(h)\right]$$

Expected free energy minimisation is now a minimisation over three parameters: the habituation *h*, the habituation statistics $\theta_{h}$, and the policy *π*. When stress is induced because the goal prior cannot be attained by any policy at hand, the risk terms are high. Any reduction of expected free energy needs to minimise risk with the constraint that $\theta_{h}$ can only be affected during habituation (*h* = 1). Note that the habituation state *h* is generic for all policies. This means that optimising the habituation state necessarily integrates expectations under all policies entertained by the agent.

In general, there will be always a trade-off for the agent to make. By habituation, the free energy is increased because the posterior *q*(*h* = 1) = 1 is significantly different from the habituation prior $p(h=1)=\hat{h}$ as long as $\hat{h}$ is relatively small, which we assume here because habituation should only be available as a last resort for the agent and not as a daily preference changing scheme. We refer to this as the habituation costs in the figures later on. On the other hand, habituation has also a decreasing effect on expected free energy because the difference between the goal prior and the posterior of the policy selection is reduced. The stronger the agent is—in its beliefs about the goal states—the higher is the pressure for habituation when no policy fulfils the goals. The smaller the habituation tendency, the higher is the pressure (in the opposite direction) for non-habituation. Being able to minimise risk (or the discrepancy between goal states and control states)—i.e., being able to influence one’s environment—is known as KL `optimality’. In previous active inference literature, the *beliefs about* ‘KL optimality’ are associated with a *sense* of agency (Friston et al., 2013). The agent’s prior experiences inform beliefs about how strongly their own actions correspond with observable effects: if there is a low correspondence (I act repeatedly but nothing comes of it), the agent’s confidence (precision) in their beliefs about being KL optimal reduces—in other words, they lose their sense of being an agent. It may be, therefore, that agents who have low confidence in their agency are more likely to change their goal prior—i.e., this may influence the habituation prior.

Outer factors (like the environment), the policies and internal factors (like the goal prior and the habituation prior) affect the habituation process. In the same scenario, where agents have the same policy options, one agent might habituate while another agent with a different habituation prior might not. As noted above, we assume that the effect of habituation on the goal prior is an effect on its precision rather than changing the overall structure. This interpretation is in line with other work on the goal prior, e.g., the initial hypothesis of Peters et al. (2017) on stress habituation and goal priors and the work by Linson et al. (2020) who use very specific goal priors to explain the results in behaviour of PTSD patients. Our general structure would also allow $\theta_{h}$ to be any other statistics of the distribution. In the next section, we will illustrate the habituation process when $\theta_{h}$ is connected to precision. Later, we discuss the physiological arguments for this.

## Stress Habituation in the Context of Work Stimulus Preferences and Social Acceptance

Above, we have already given some examples of high-level goal priors being involved in causing stress. In this section, we introduce the preference for work stimulus and the need for social acceptance as further examples that can result in stress. The phenomenon when work becomes too much or too complex and the agent cannot find a way out of the situation is discussed extensively in the media and in research as burnout (Maslach and Leiter, 2006; Bang and Reio, 2017). Burnout is often connected with overwhelming demands that cannot be fulfilled (Maslach et al., 2001; Fralick and Flegel, 2014). Another source of stress can also be the situation when work is boring and is discussed under the term bore-out (Schaufeli and Salanova, 2014; Stock, 2015).

Here, we consider a scenario where the agent has to decide either to take a job that is very demanding and exceeds one’s skills and work stimulus, or to keep the current job, which is very repetitive and boring. In our scenario, no other job offer is currently available, and in both situations, there is some deviation from the agent’s preference for work stimulus.

**Figure 1:**
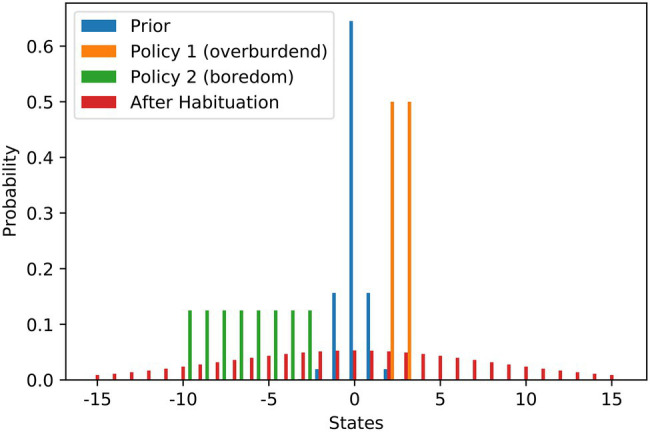
Work stimulus scenario—flattening of probability distributions over goal states underlying stress habituation. The bars show the different probability distributions used in the example of work stimulus preference. The blue distribution is the prior distribution that is encoding the agent’s preferences before habituation. The orange and green distributions visualise the states that can be attained with the two strategies at hand. The orange policy is connected to a scenario of an overwhelming work stimulus, while the green distribution is connected to a scenario of staying in a repetitive and boring job. The red distribution shows the optimal prior distribution the agent would choose if he could optimise its own prior. In our scenario, the red distribution can only be installed as a new prior by habituation.

We model the scenario as a discrete setup and use states from −15 to 15 to denote the work stimulus (we use it just as an ordinary scale where 0 is in the middle). Figure 1 contains the probability distributions used in the modelling of the scenario. We set the most preferred work stimulus as the value 0. To model the initial goal prior (blue), we use a discrete variant of the Gaussian distribution, that is rescaled to a sum of 1 to be a valid probability distribution. The variance of the prior is relatively low resulting in a steep peak at *S = 0*. We use the variance of the distribution as the habituation parameter $\theta_{h}.$ The agent has two policies to choose from. With the demanding job (orange), a higher work stimulus of 2 or 3 is attainable, while with the current repetitive job (green), only stimulus values between −10 and −3 can be attained. Both policies result in high levels of expected free energy due to the large risk term. Before any habituation, taking the demanding job offer is the free energy optimal choice. When the agent becomes habituated, the goal prior would be changed to the flat distribution (red) *via* increasing the variance $\theta_{h}$. After habituation, flattening of the goal prior results in a shift of the best policy to choose, which is then keeping the repetitive job. As mentioned above, habituation also comes at a cost that is depending on the agent’s habituation tendency $\hat{h}.$ Only when the benefits of changing the goal prior outweigh the costs of habituation does the agent actually become habituated and change their policy decision.

**Figure 2:**
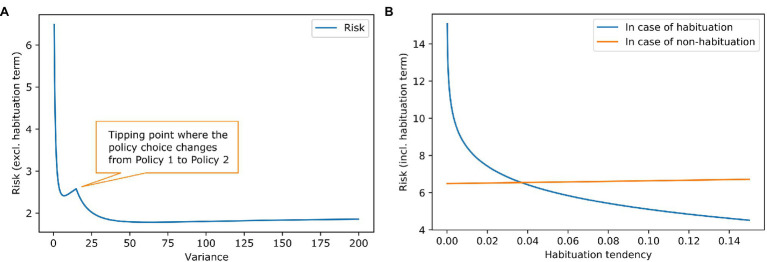
Work stimulus scenario—effect of goal prior variance and habituation tendency on risk. **(A)** The blue line shows the risk term (excluding the habituation costs) dependent on the variance of the goal prior. We exclude the cost of habituation to show the influence of the goal prior shape on the policy choice. It can be seen, that risk is reduced if the goal prior is progressively flatter, because of the high mismatch between goal prior and attainable states in the policies. There is a tipping point where the policy choice for the agent changes. **(B)** The lines show the complete risk values (including the habituation costs) in case of habituation (blue) and in case of no habituation (orange) dependent on the habituation tendency. It is clear that risk for habituation gets smaller when the habituation tendency gets higher because the penalty for habituation is reduced. The orange line remains relatively constant which is intuitive because in case of no habituation the goal prior is not affected, and the penalty is not reduced. However, there is a slight increase in risk. This is due to our assumption that the agent strictly infers to not habituate. Before the inference, the agent had a (very) small preference for habituation which can be interpreted as a small value for keeping the option open. In the posterior *q*(*h*) though, the option is gone [in case of no habituation, it is *q*(*h*) = 0]. A higher habituation tendency $\hat{h}$ means a higher value for the option to habituate thus resulting in an increase of the orange line, where this option is gone.

Figure 2A shows the risk term (excluding the habituation costs) as a function of the goal prior variance. It can be seen that there is a local minimum at a variance of about 7, where the agent still prefers the policy of accepting the challenging job offer. At a variance of about 15, there is a tipping point. After that, it is more attractive to keep the repetitive job than to aim for the demanding job and risk too high a work stimulus. The global minimum is somewhere around 60. A higher than 60 variance increases the risk term, but only very slightly.

Knowing that some agents habituate to stress and others do not, we examine different values for habituation tendency. Figure 2B contains the calculation of the full risk term (including the habituation costs) depended on the habituation tendency. When the habituation tendency is less than ~0.04, the risk term for habituation is higher than for non-habituation because the costs of habituation outweigh its benefits; in this case, non-habituation would be the better choice. When the habituation tendency is higher than ~0.04, the benefits of habituation outweigh its costs, making habituation the better choice. Using the free energy principle, a difference in a high-level prior characterising habituation tendency could explain why some people benefit from habituation and others do not.

The example shows that the discrete effect of habituation and non-habituation can be explained by the greater propensity of habituators to change goal priors and the lesser propensity of non-habituators to do so. The tendency to habituate is modelled as a binary prior distribution that is specific to context and to the agent (Kirschbaum et al., 1995). In the two-policy job choice scenario, the habituator chooses to stay in the repetitive-unfulfilling situation, while the non-habituator maintains his steep goal and takes the demanding, high-pressure job despite the stress it entails. For the non-habituator, the stress of being underchallenged is more pronounced (higher free energy) than that of accepting the challenging work. Due to the low habituation tendency, the non-habituator is only in rare cases able to broaden their goal preferences and thus to get used to difficult life situations.

**Figure 3:**
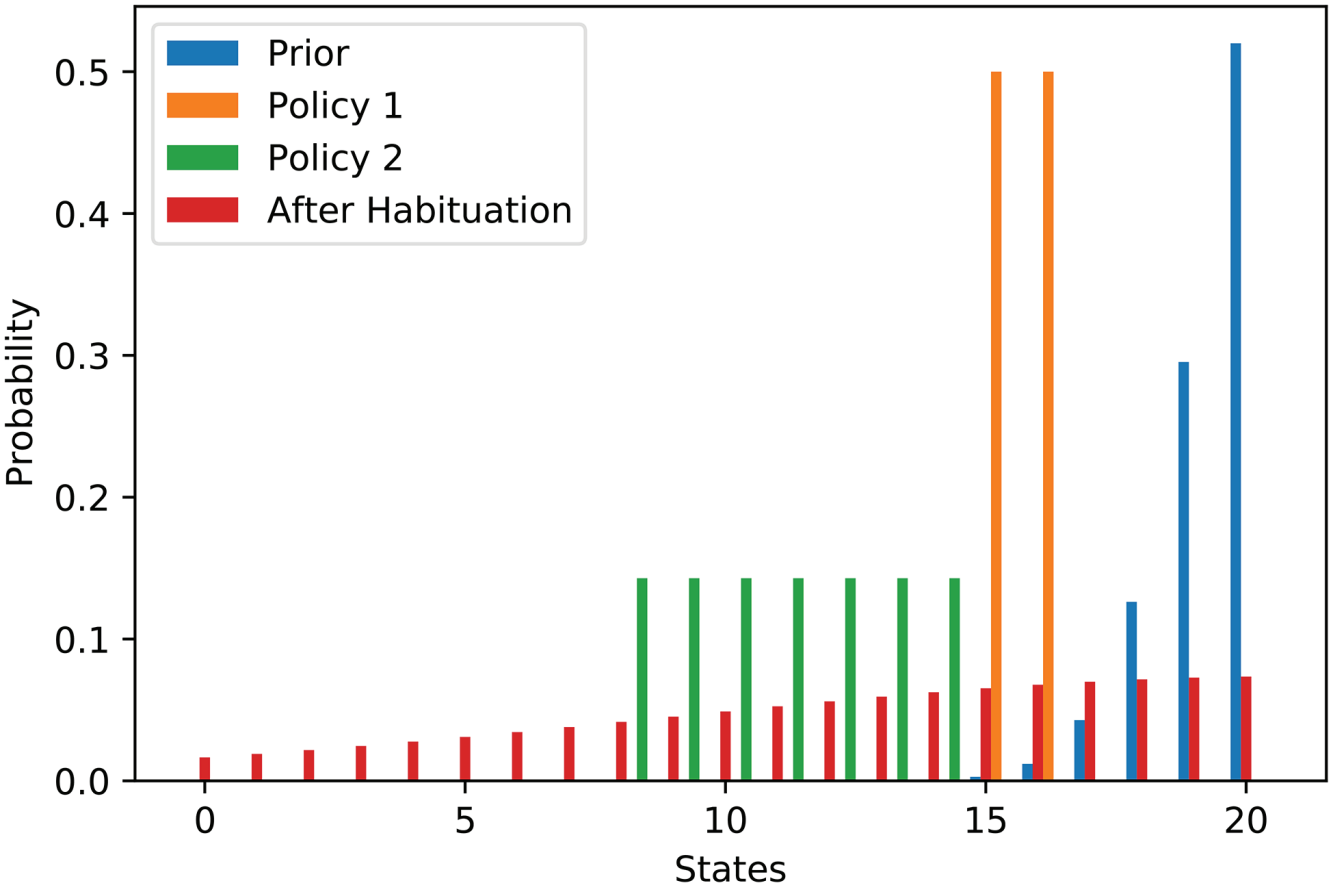
Social acceptance scenario—flattening of probability distributions over goal states underlying stress habituation. The bars show the different probability distributions used in the example of social acceptance analogously to the example of work stimulus. Here, the most preferred situation is to have as much social acceptance as possible.

The work stimulus example is one where the agent has its maximum preference somewhere within the state spectrum, e.g., in the middle. Of course, other goal priors are also conceivable. There are a number of situations in which the principle of ‘the more the better’ applies to preferences, with the optimal preference at one end of the state spectrum. Examples could be wealth or social acceptance. We use the left part of a discrete bell curve that we normalised to sum-up to one to illustrate the preference for social acceptance. Similar to the previous example, Figure 3 contains the probability distributions. In this example, the agent has a clear preference (blue) to arrive on the right end of the state spectrum, i.e., at level 20. The agent’s first policy (orange) allows them to achieve a level of social acceptance of 15 or 16, while the second policy (green) has a broader spectrum of states, ranging from 8 to 14. Both policies are afflicted with a high degree of stress due to their high-risk terms.

**Figure 4:**
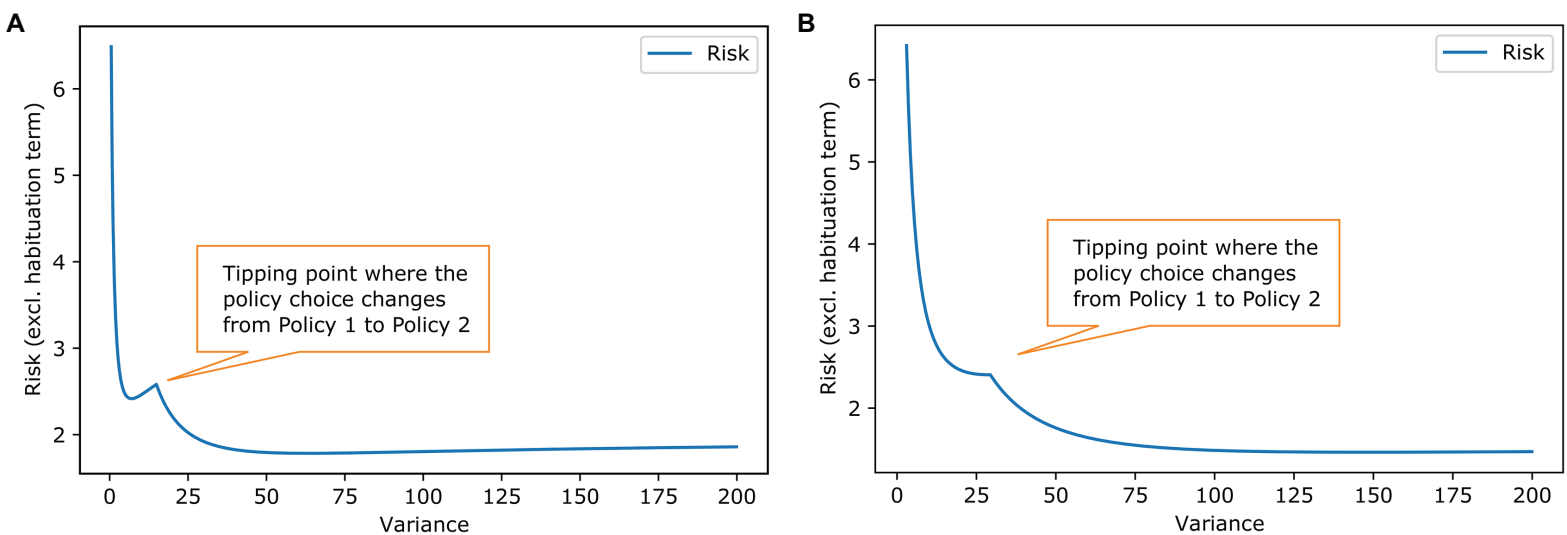
Social acceptance scenario-effect of goal prior variance and habituation tendency on risk. The plotted lines work analogously to the lines in Figure 2 in the example of work stimulus. **(A)** The blue line shows again the influence of the prior shape. The tipping point for choosing another policy has slightly different shape but results in the same conclusion as in Figure 2 that a habituating agent will change its policy choice. **(B)** The liens are anologue to Figure 2B. Here, a habituation tendency above roughly 0.03 results in stress habituation of the agent.

Figure 4 shows the habituation analyses from Figure 2 for the social acceptance scenario. The second example shows that different forms of habituation priors are possible. There has been some literature on Gaussian shaped priors (Friston, 2009), but in general, our model is agnostic to the prior’s form as long as we have sufficient statistics that can be influenced by the stress habituation. In both examples, the habituating agent changes their best policy. This is chosen by design to show how habituation can change behaviour, but it does not have to be the case in all scenarios. Stress habituation can also occur if the policy choice stays the same; then, it is just a vehicle for the agent to reduce their stress level. From the outer perspective, no change in behaviour can be seen, but the agent no longer shows stress arousals in the context to the homotypic stressor.

Comparing the overall stress of habituated and non-habituated individuals, habituated agents display much less free energy, i.e., they suffer from less stress than non-habituated individuals (see Figure 2A). While agents who habituated are characterised by the elimination of stress arousals on certain homotypic stressors, agents who did not habituate show continuous or intermittent stress arousals, even at night when they become insomniac. As a buffer mechanism, habituation can lead to ‘tolerable stress’ (Peters et al., 2017). In fact, these people show only low cortisol responses and intermediate levels of self-esteem and locus of control (Kirschbaum et al., 1995; Pruessner et al., 1999, 2005). When buffer mechanisms such as habituation fail and individuals remain trapped in their aversive environment, ‘toxic stress’ can result; the stress responses of these individuals are maximal, while their self-esteem and sense of control are minimal (Kirschbaum et al., 1995; Pruessner et al., 1999, 2005). Thus, habituation is a means of escaping the harmful effects of toxic stress. But the price of habituation is that the habituated person has to give up their deepest preferences.

## Consequences of Stress Habituation

Habituation can affect both the internal body states of the agent and the external states that the agent occupies. The internal body states include the energy states of both brain and body. As we have shown so far, habituation minimises the complexity term in the free energy formulation but interestingly also minimises the cerebral metabolic cost. For the complexity, costs of variational free energy are related to the brain’s metabolic costs (i.e., the thermodynamic free energy) in the sense that they have the same minimum (Sengupta et al., 2013). Thus, statistically and metabolically efficient brains penalise high complexity and associated commodities like energy. With respect to habituation, this means that reducing the precision of goal priors may reduce not only complexity costs but also cerebral metabolic costs.

These theoretical considerations on the close relationship between variational and thermodynamic free energy made in previous work (Sengupta et al., 2013; Kiefer, 2020; Parr et al., 2020) are consistent with the experimental evidence from stress research. Experimentally induced stress arousals (i.e., states with high variational free energy) have been shown to be highly energetically costly; even mild mental laboratory stressor results in a 12% increase in global cerebral metabolic rate of glucose (Madsen et al., 1995). Occasional arousal states are part of every vibrant and good life. People with toxic stress show recurrent or persistent arousal states during the day and insomnia at night, which increases the average brain’s energy need. Compared to people who live a vibrant and good life, people who have habituated to stress hardly show any arousals, so their average brain energy need is lower.

**Figure 5:**
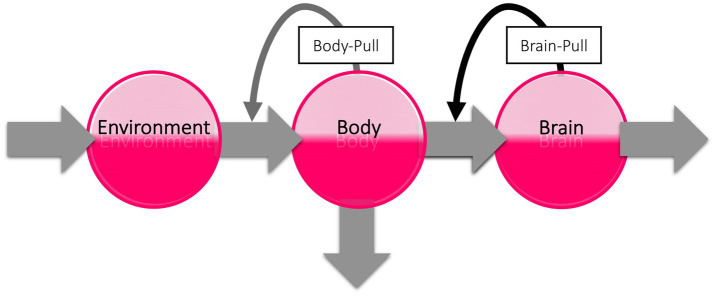
The cerebral supply chain. Energy is transferred from the environment through the body to the brain, the end consumer of the supply chain. When the brain needs energy, the brain-pull demands it from the body. When the body needs energy, the body-pull demands it from the environment.

The subaverage cerebral energy demand and need after habituation may have a major impact on peripheral energy metabolism. To illustrate this, we take a look at the brain’s energy logistics. As we have shown in three recent systematic reviews, the brain can be viewed as the end consumer of an energetic supply chain (Figure 5; Sprengell et al., 2021a,b,c). The cerebral supply chain is a mathematical representation of the Selfish Brain theory. The Selfish Brain theory describes the mammalian brain’s ability to regulate energy metabolism in such a way that it covers its own high need with priority (Peters et al., 2004). The brain behaves ‘selfishly’ in this respect. The key feature of the Selfish Brain theory is the postulate of brain-pull mechanisms by which the brain procures itself with ‘energy on demand’ (Peters and Langemann, 2009). Rival theories are the gluco-lipostatic theory and its modern variants denying the existence of brain-pull mechanisms (Kennedy, 1953; Mayer, 1953; Chaput and Tremblay, 2009; Schwartz et al., 2017). In the three systematic reviews mentioned above, the competing theories were put to the test. The Selfish Brain theory was able to predict all the data sets studied (i.e., on caloric restriction, cerebral artery occlusion, and type 1 diabetes mellitus), whereas all predictions of the gluco-lipostatic theory and its modern variants failed (Sprengell et al., 2021a,b,c). Based on the current evidence, we can therefore rely on the cerebral supply chain model for our further considerations of stress habituation.

According to the general laws of supply chains, goods build up when the end user needs and demands less (Slack et al., 2004). If customers do not buy, the shelves stay full. This is exactly what can be expected when the brain consumes less energy after stress habituation, namely, that energy builds up in the body, leading to weight gain. The opposite occurs with toxic stress, when the brain consumes more energy and the body stores are emptied, resulting in weight loss. Because in toxic stress, peripheral glucose storage is inhibited, glucose can accumulate in the blood, so that the lean type 2 diabetes phenotype develops. However, when after stress habituation the brain consumes less energy, the energy accumulates in the adipose tissue (in the form of triglycerides) and in the blood (in the form of glucose), resulting in the obese type 2 diabetes phenotype (Peters and McEwen, 2015). In short, when the brain, as the end consumer, needs less energy after stress habituation, energy builds up in the cerebral supply chain—leading to obesity.

At first glance, one might expect that behavioural changes under toxic stress and after habituation would also contribute to changes in body shape. Under toxic stress, social withdrawal may occur due to depression, and after habituation, submissive, withdrawn, and undynamic behaviour may develop, so in both cases, decreased physical activity can be expected to cause weight gain. But this is unlikely to be the case, as toxic stress is more related to weight loss and habituation to weight gain (Epel et al., 2000; Peters and McEwen, 2015), making such behavioural factors on body shape appear less contributing.

The external states that the agent occupies may also change through the habituation process. Looking at a population of people who have habituated to homotypic stressors (compared to non-habituated people), these people are more likely to occupy adverse (previously unaccepted) states (e.g., lower social status and poverty). This phenomenon is due to the preferences that have been established after habituation, which are less precise and thus make adverse states seem more acceptable. Thus, people who have habituated are unlikely to continue searching for promising ways out.

Interestingly, one could infer from our theoretical considerations that stress habituation is a risk factor on the one hand for the development of obesity and on the other hand for the tolerance/acceptance of low socio-economic status. Such relationships could explain, at least to some extent, why many people of low socio-economic status are obese (Mohammed et al., 2019). Further evidence comes from the MTO project, a large social intervention study in which women in the intervention arm were able to move from a poverty area to a better vicinity. Compared to the control group, the women who were given the opportunity to move felt less uncertain and had a lower prevalence of severe obesity and type 2 diabetes after 15 years (Ludwig et al., 2011, 2012). These findings are consistent with the notion that alleviating stress through social interventions reduces the prevalence of obesity, a notion strongly supported by findings from animal experiments which demonstrated that stress causes obesity (Kaufman et al., 2007; Jahng et al., 2012). It is tempting to speculate that in the MTO project, less stress among the women who moved led to fewer stress habituations and thus to a lower prevalence of obesity.

Given the presence of heterotypic stressors in a complex environment, a person who has habituated to one stressor (e.g., at work) may not habituate to another (e.g., at home). Thus, complex interactions between the individual and their environment determine the extent to which internal and external consequences of habituation manifest themselves.

In summary, stress habituation based on less precise goal preferences, on the one hand, makes stress more tolerable (by reducing variational free energy to some extent) and protects against the deleterious effects of toxic stress, but, on the other hand, makes the occupation of precarious living conditions and the development of the obese type 2 diabetes mellitus phenotype more likely.

## Conclusion and Outlook

Three arguments support the notion that our approach captures the nature of stress habituation well:

1. The free energy optimising perspective is a very general perspective that has been used to explain various behaviours. Stress habituation can easily be integrated into the free energy principle, which makes our approach plausible because of the mathematical unification.
2. Stress habituation has been found to occur in brain areas where goal priors are encoded (vmPFC). In addition, it could be shown that in the vmPFC, the goal preferences are coded as the mean and variance of the internal probability distributions. Given this evidence, we propose that stress habituation decreases the precision (inverse variance) of goal preferences. The vmPFC in turn controls stress responses (amygdalae), which makes our approach plausible from an anatomical-functional point of view.
3. The mathematics behind the optimisation are well defined and can lead to easy-to-understand examples, as shown in Section “Stress Habituation in the Context of Work Stimulus Preferences and Social Acceptance”.

In summary, given the currently available theoretical and experimental background, our novel approach (compared to the conventional one) provides the best explanation. Future human studies combining repeated TSSTs with dynamic causal models could provide further insights into the mechanisms of stress habituation.

## Author Contributions

AP developed the conceptual idea. MH created the examples. All authors contributed to the article and approved the submitted version.

## Conflict of Interest

MH is employed by singularIT GmbH.

The remaining authors declare that the research was conducted in the absence of any commercial or financial relationships that could be construed as a potential conflict of interest.

## Publisher’s Note

All claims expressed in this article are solely those of the authors and do not necessarily represent those of their affiliated organizations, or those of the publisher, the editors and the reviewers. Any product that may be evaluated in this article, or claim that may be made by its manufacturer, is not guaranteed or endorsed by the publisher.

## Footnotes

^1^For example, by considering new policies—or nuancing the balance between prior beliefs and sensory evidence, through changing their relative precision. Physiologically, this corresponds to a neuromodulatory change in the excitability of gain of neuronal message passing. In turn, this is manifest as a change in synaptic rate constants, i.e., rates of evidence accumulation associative plasticity. Psychologically, this is generally thought of in terms of selective attention and sensory attenuation and concomitant experience-dependent plasticity.
